# Supplementary material for: Spatiotemporal dynamics of tumor–CAR T-cell interaction following local administration in solid cancers
Source: PLoS Comput Biol. 2025 Jun 3;21(6):e1013117. doi: 10.1371/journal.pcbi.1013117 (PMC12165384; doi:10.1371/journal.pcbi.1013117)
Supplement: S1 Text — contains further details on the mathematical model and numerical simulations used to generate the results in this manuscript, as well as the following supporting figures and tables of parameter values.Fig A. Model-predicted tumor volume doubling time (VDT) and tumor burden at detection as a function of tumor growth parameters.Fig B. Exhausted cells improve model fit to CAR T-cell data from mouse imaging study.Fig C. Outcome maps given varied tumor size at the time of treatment.Fig D. Outcome maps given varied time of patient evaluationFig E. Relating quantitative tumor characteristics to minimum CAR T-cell dose for tumor eradicationFig F. Convergence analysis via method of manufactured solutionsTable A. Parameter values used in numerical simulations of the 4 tumor types described in Section 2.2Table B. Estimated model parameters to fit model output to murine study data (PDF) [file pcbi.1013117.s001.pdf]

Supporting information for

Spatiotemporal dynamics of tumor-CAR T-cell interaction following local administration in solid cancers

Katherine Owens[1,2], Aminur Rahman[1,3], and Ivana Bozic[1,4*]

[1] *Department of Applied Mathematics, University of Washington, Seattle, Washington, United States of America*
[2] *Vaccine and Infectious Disease Division, Fred Hutchinson Cancer Center, Seattle, Washington, United States of America*
[3] *Artificial Intelligence Institute in Dynamic Systems, University of Washington, Seattle, Washington, United States of America*
[4] *Public Health Sciences Division, Fred Hutchinson Cancer Center, Seattle, Washington, United States of America*

Ivana Bozic
email: ibozic@uw.edu

This PDF file includes:

- Supporting text
- Figures A to F
- Tables A and B
- SI References

S1 Nondimensionalization

We nondimensionalize Eq. (5) with $\tau = at$, $\hat{r} = r/R_0$, $\hat{u} = bu$, and $\hat{v} = bv$, where a is the tumor proliferation rate from [1], R_0 is the initial spread of the tumor, and $1/b$ is the maximum tumor cell density. This gives us the equation (with hats removed)

$$\frac{\partial u}{\partial t} = \frac{1}{r^2}\frac{\partial}{\partial r}\left(D_T(u) r^2 \frac{\partial u}{\partial r}\right) + F_1(u,v); \qquad D(u) = \begin{cases} 0 & u(r,t) \le \hat{u}^*, \\ D_T^* & u(r,t) > \hat{u}^*; \end{cases} \tag{S1a}$$

$$\frac{\partial v}{\partial t} = \frac{1}{r^2}\frac{\partial}{\partial r}\left(D_C r^2 \frac{\partial v}{\partial r}\right) + F_2(u,v). \tag{S1b}$$

with

$$F_1 = \left[1 - u - \gamma \frac{v^l}{su^l + v^l}\right] u \tag{S2a}$$

$$F_2 = \alpha \left[\frac{v^{2l}u^2}{(su^l + v^l)^2 + \zeta_C v^{2l} u^2} - \beta_C u - \chi\right] v \tag{S2b}$$

where $\hat{u}^* = bu^*$, $\alpha = d^2 j/kab^2$, $\beta_C = kqb/d^2 j$, $\gamma = d/a$, $\chi = mkb^2/d^2 j$, and $\zeta = d^2/kb^2$.

S2 Numerical Methods

Finite difference schemes are employed to solve the radially symmetric spherical diffusion equations in the model (4). Namely, Crank-Nicolson (CN) [2] is used for the results presented in the body of the paper. For the concentration of CAR T cells, $v_m^n = v(m\Delta r, n\Delta t)$, the CN scheme is fairly standard yielding

$$\begin{aligned} v_m^{n+1} - v_m^n &- \frac{D_C}{r_m}\left[v_{m+1}^{n+1} - v_{m-1}^{n+1} + v_{m+1}^n - v_{m-1}^n\right]\frac{\Delta t}{2\Delta r} \\ &- D_C\left[v_{m+1}^{n+1} - 2v_m^{n+1} + v_{m-1}^{n+1} + v_{m+1}^n - 2v_m^n + v_{m-1}^n\right]\frac{\Delta t}{2(\Delta r)^2} \\ &- \left[F_2\left(u_m^{n+1}, v_m^{n+1}\right) + F_2\left(u_m^n, v_m^n\right)\right]\frac{\Delta t}{2} = 0, \end{aligned} \tag{S3}$$

with $v_{m+1} = v_{m-1}$ at $r = 0$ and for $r \in \partial\Omega(u)$

For the tumor concentration, $u_m^n = u(m\Delta r, n\Delta t)$, the diffusivity, D_T, depends on u, which means its dependence on r is changing in time. If u_{m+1}^n and u_{m-1}^n are both larger than $\hat{u}^*$ or both less than $\hat{u}^*$, the central difference for D_T is trivial, and hence D_T is treated as a constant. However, if the parity is different, there is no flux from the side with a concentration less than $\hat{u}^*$, and therefore it can be treated as a Neumann boundary. If u_{m+1}^n and u_{m-1}^n have the same parity relative to $\hat{u}^*$ we write

$$\begin{aligned} u_m^{n+1} - u_m^n &- \frac{D_T}{r_m}\left[u_{m+1}^{n+1} - u_{m-1}^{n+1} + u_{m+1}^n - u_{m-1}^n\right]\frac{\Delta t}{2\Delta r} \\ &- D_T\left[u_{m+1}^{n+1} - 2u_m^{n+1} + u_{m-1}^{n+1} + u_{m+1}^n - 2u_m^n + u_{m-1}^n\right]\frac{\Delta t}{2(\Delta r)^2} \\ &- \left[F_1\left(u_m^{n+1}, v_m^{n+1}\right) + F_1\left(u_m^n, v_m^n\right)\right]\frac{\Delta t}{2} = 0, \end{aligned} \tag{S4}$$

otherwise we write

$$u_m^{n+1} - u_m^n - D_T\left[u_\pm^{n+1} - u_m^{n+1} + u_\pm^n - u_m^n\right]\frac{\Delta t}{(\Delta r)^2} - \left[F_1\left(u_m^{n+1}, v_m^{n+1}\right) + F_1\left(u_m^n, v_m^n\right)\right]\frac{\Delta t}{2} = 0, \tag{S5}$$

where $u_\pm = u_{m+1}^n$ if $u_{m+1}^n > \hat{u}^*$ or $r_m = 0$, and $u_\pm = u_{m-1}^n$ if $u_{m-1}^n < \hat{u}^*$.

The use of Crank-Nicolson finite difference methods on an equation with a step-function diffusivity is made possible due to the radial symmetry of the domain. For more complex geometries it will be necessary to employ finite element methods, which we leave for a future study.

S2.1 Boundary and Initial Conditions

In this section consider $\partial\Omega(u) = 4\max(R_{\text{tumor}})$ to be the boundary of the region of influence of the tumor Ω, where R_{tumor} are the radial values such that $u > 0$; that is, only active CAR T cells within the influence of the tumor region are considered since most CAR T cells remain near the tumor.

Since diffusion is the main driver of the dynamics before cell-to-cell interactions begin, we consider an initial profile with a compact Gaussian shape (i.e., a bump function) shortly after injection. The bump function assumption simplifies the model by obviating the dynamics of the injection itself. Both the internal and external injection bump functions are illustrated in Fig 1.

At the boundary, $\partial\Omega$, we consider a Neumann condition

$$\frac{\partial u}{\partial r} = \frac{\partial v}{\partial r} = 0, \tag{S6}$$

since we keep our boundary away from the tumor and we want to prevent precipitous leakage of the CAR T cells away from the tumor microenvironment. To implement the condition at the boundary, we use ghost points, where for the purpose of the centered difference, we assume just past the boundary and just before the boundary the concentration is equivalent. That is, if $m = M$ corresponds to $r = \partial\Omega$, then $u_{M+1} = u_{M-1}$ and $v_{M+1} = v_{M-1}$. This yields the equations

$$u_M^{n+1} - u_M^n - D_T\left[u_{M-1}^{n+1} - u_M^{n+1} + u_{M-1}^n - u_M^n\right]\frac{\Delta t}{(\Delta r)^2} - \left[F_1\left(u_M^{n+1}, v_M^{n+1}\right) + F_1\left(u_M^n, v_M^n\right)\right]\frac{\Delta t}{2} = 0, \tag{S7a}$$

$$v_M^{n+1} - v_M^n - D_T\left[v_{M-1}^{n+1} - v_M^{n+1} + v_{M-1}^n - v_M^n\right]\frac{\Delta t}{(\Delta r)^2} - \left[F_1\left(u_M^{n+1}, v_M^{n+1}\right) + F_1\left(u_M^n, v_M^n\right)\right]\frac{\Delta t}{2} = 0. \tag{S7b}$$

S2.2 Manufactured Solution

Now let us consider the convergence properties using the manufactured solution

$$\tilde{u}(r,t) = \frac{1}{r}e^{-D_T^* t}\sin(r) \tag{S8a}$$

$$\tilde{v}(r,t) = \frac{1}{r}e^{-D_C t}\sin(r) \tag{S8b}$$

to test $D_T(u) = D_T^*$ This yields the test PDEs

$$\frac{\partial u}{\partial t} = \frac{1}{r^2}\frac{\partial}{\partial r}\left(D_T^* r^2 \frac{\partial u}{\partial r}\right) + F_1(u,v) - F_1(\tilde{u},\tilde{v}); \tag{S9a}$$

$$\frac{\partial v}{\partial t} = \frac{1}{r^2}\frac{\partial}{\partial r}\left(D_C r^2 \frac{\partial v}{\partial r}\right) + F_2(u,v) - F_2(\tilde{u},\tilde{v}) \tag{S9b}$$

We employ the Crank-Nicolson scheme from Sec. 4.3 on the test PDE (S9) to get the approximate solution $u(r,t)$ and $v(r,t)$. Then using the ground truth (S8), we find the absolute errors $|u(r,t) - \tilde{u}(r,t)|$ and $|v(r,t) - \tilde{v}(r,t)|$. In Fig F, we illustrate the error in practice and the convergence properties of the method for our setup. As our PDE, and therefore the test PDE as well, has a sharp boundary between the proliferative and diffusive regimes in addition to significant nonlinearities, we would not expect our implementation of the Crank-Nicolson finite difference scheme to have the same convergence properties as that of the standard diffusion equation. Even still, as we observe in Fig F, the error is quite low especially near the center of the domain where it is nearly 10^{-4}. Towards the boundary of the computational domain the error does increase to slightly greater than 1%, however we can mitigate this issue by expanding our computational domain to keep the tumor away from the boundary. As we expect, the convergence properties away from the boundary are closer to that of the standard diffusion equation with local order of convergence of $o\left(\Delta t^{1.5}, \Delta r^{1.1}\right)$ and $o\left(\Delta t^1, \Delta r^{0.6}\right)$ for u and v respectively.

S3 Supplementary Figures

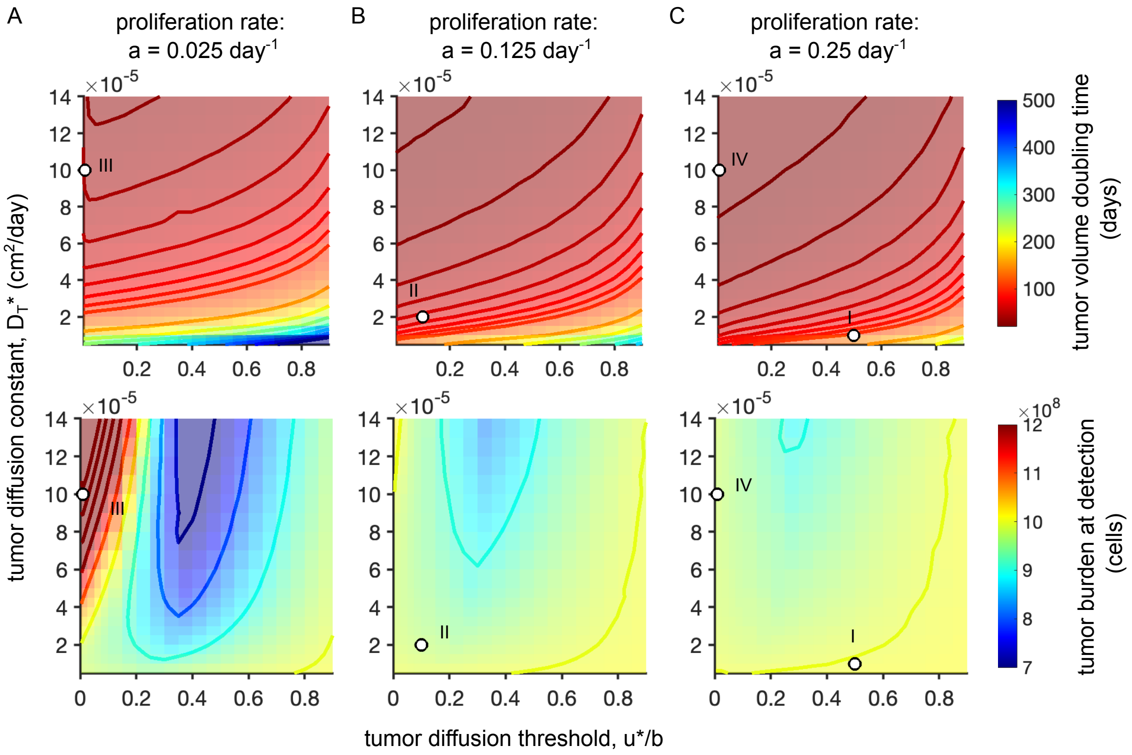

Figure A: *Model-predicted tumor volume doubling time (VDT) and tumor burden at detection as a function of tumor growth parameters.* In each column a fixed value was used for the tumor cell proliferation rate, while the tumor diffusion constant was varied from $5e-6$ to $1.4e-4$ cm^2/day and the threshold tumor density governing the onset of diffusion was varied from 1-90% of the tumor density carrying capacity. Panel A shows tumor behavior at a low proliferation rate of $a = 0.025$ day^{-1}, panel B a medium proliferation rate of $a = 0.125$ day^{-1}, and panel C a high proliferation rate, $a = 0.25$ day^{-1}. For each simulation we calculated VDT (top row) by recording the time a spherical tumor grew from a detectable radius of 1 cm to a detectable radius of $2^{1/3}$ cm. We also calculated the tumor burden at detection by integrating the tumor cell density across the spatial domain to get total cell count when the detectable diameter was 2 cm. The tumor growth parameters defining tumor types I-IV are indicated by white circles on the appropriate panel.

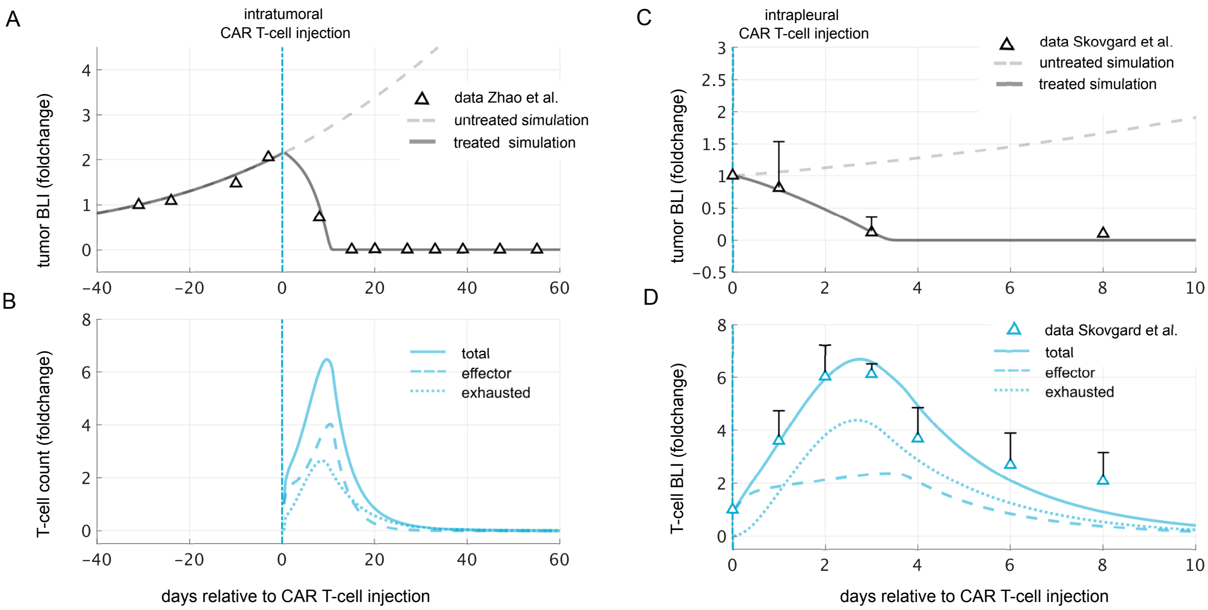

Figure B: *Exhausted cells improve model fit to CAR T-cell data from mouse imaging study.* As in Fig 5 in the main text, panels (A) and (B) show simulation results comparing tumor outcomes with data from an experiment in which Zhao et al. established large flank mesothelioma tumors in mice, measured baseline tumor size using bioluminescent imaging (BLI), injected 1×10^7 CAR T cells intratumorally 58 days following tumor establishment, and continued to measure the tumor size using BLI for 55 days. In this case the effector and exhausted CAR T-cell populations are shown separately and in total in panel (B). Panels (C) and (D) show simulation results comparing tumor and CAR T-cell kinetics against data from Skovgard et al., who established small antigen-positive orthotopic mesothelioma tumors in mice, measured baseline tumor size using bioluminescent imaging (BLI), injected 1×10^7 CAR T cells intrapleurally 14 days following tumor establishment, and continued to measure the tumor size and CAR T-cell quantity for 8 days using BLI. Panel (D) shows the fraction of CAR T cells that are in the effector and exhausted states over time, along with the simulated total.

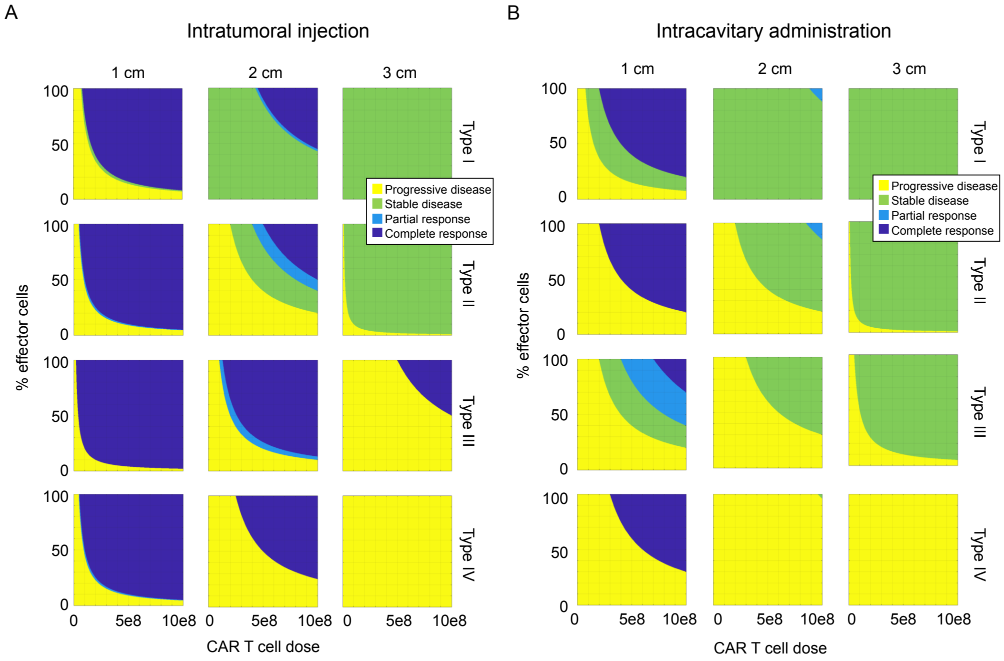

Figure C: *Outcome maps given varied tumor size at the time of treatment.* We simulated (A) intratumoral injection and (B) intracavitary administration of CAR T cells to treat tumors with varied growth parameters and initial sizes. Within each subfigure, the row corresponds to a different tumor type as defined in section 4.5, with type I having the longest volume doubling time and type IV having the shortest volume doubling time. The columns correspond to a different tumor radius at the time of treatment with column 1 being 1 cm, column 2 is 2 cm and column 3 is 3 cm. Each panel maps a range CAR T-cell doses and percentage of non-exhausted cells within that dose to patient outcome, classified using the RECIST criteria[3] at 8 weeks post-treatment.

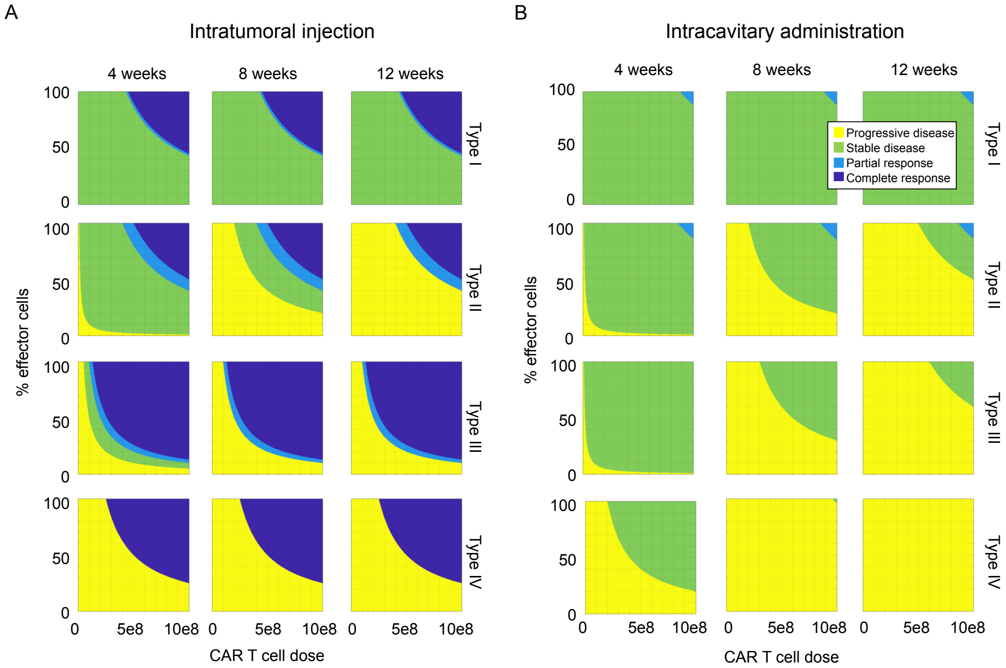

Figure D: *Outcome maps given varied time of patient evaluation.* We simulated (A) intratumoral injection and (B) intracavitary administration of CAR T cells to treat varied tumor types. Within each subfigure, the row corresponds to a different tumor type as defined in Table A and illustrated in Fig 2, with type I having the longest volume doubling time and type IV having the shortest volume doubling time. The columns within each subfigure correspond to a different date of evaluation, with column 1 being evaluation at 4 weeks, column 2 at 8 weeks, and column 3 at 12 weeks post-treatment. Each panel maps a range CAR T-cell doses and percentage of non-exhausted cells within that dose to patient outcome. In each simulation, CAR T-cell treatment occurred when the detectable tumor radius reached 2 cm.

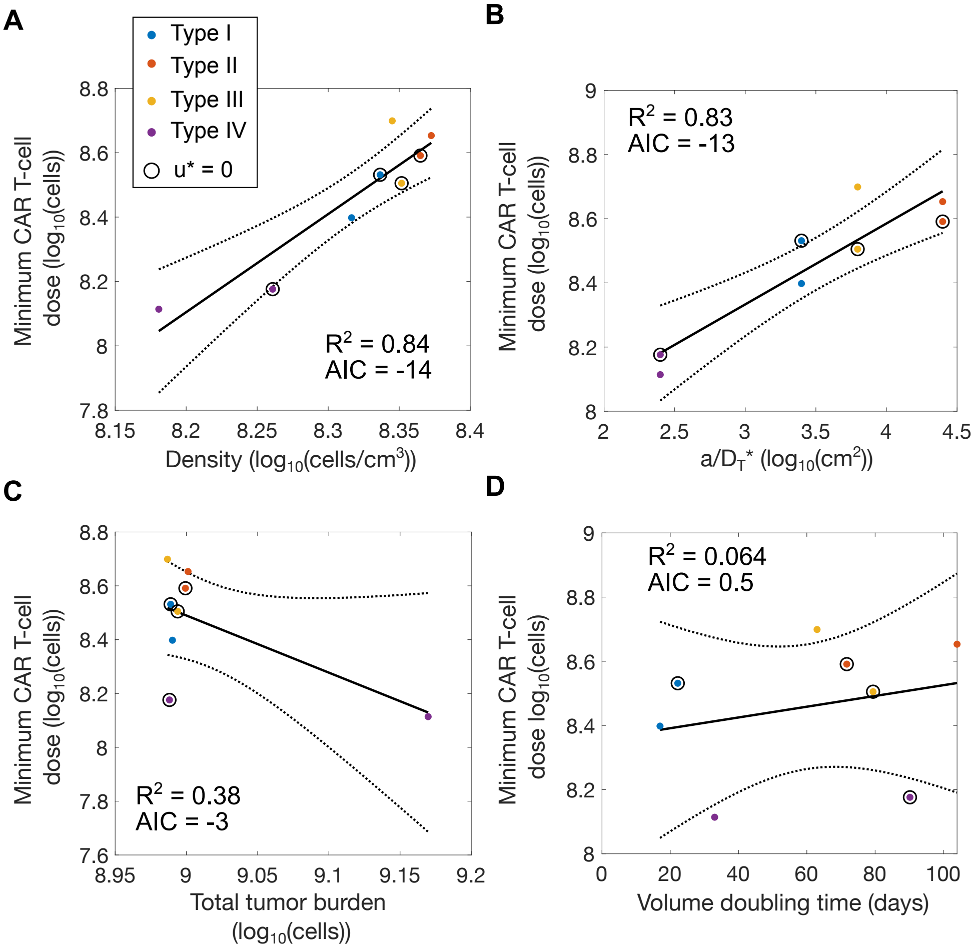

Figure E: *Relating quantitative tumor characteristics to minimum CAR T-cell dose for tumor eradication.* For each of the four tumor cell archetypes defined in Table A and illustrated in Fig 2, we established the minimum CAR T-cell dose that eradicates a tumor with detectable radius of 2 cm when delivered intratumorally with a ratio of 100% effector CAR T cells. We also repeated the same process, setting the tumor diffusion threshold $u^* = 0$ to ensure that results are not an artifact of thresholding diffusion. In each panel, the solid black line shows a best-fit linear model and the dashed lines show 95% confidence bounds calculated in matlab using the fitlm() function. For each linear model, we calculated the coefficient of determination, R^2, and Akaike Information Criterion, AIC, for comparison. (A) The average density of the detectable portion of each tumor type at the time of treatment has the highest R^2 value and lowest AIC score, indicating that this is the best predictor of the minimum CAR T-cell dose for successful treatment among the quantities considered here. (B) The ratio of the tumor proliferation rate a to the diffusion constant D_T^* has an AIC score within 2 of the tumor density AIC score, indicating that this is an equally good predictor for these tumor profiles. (C) Assuming a fixed detectable radius of 2 cm at the time of treatment, the total tumor burden is a significantly worse predictor of treatment success. Total tumor burden is calculated by integrating the tumor cell density across the spatial domain to get total cell count. Note that if the detectable radius is *not* fixed, then total tumor burden is correlated with the mimimum effective dose as shown in Fig C. (D) Volume doubling time, the time a spherical tumor grew from a detectable radius of 1 cm to a detectable radius of $2^{1/3}$ cm, is not correlated with the minimum successful CAR T-cell dose.

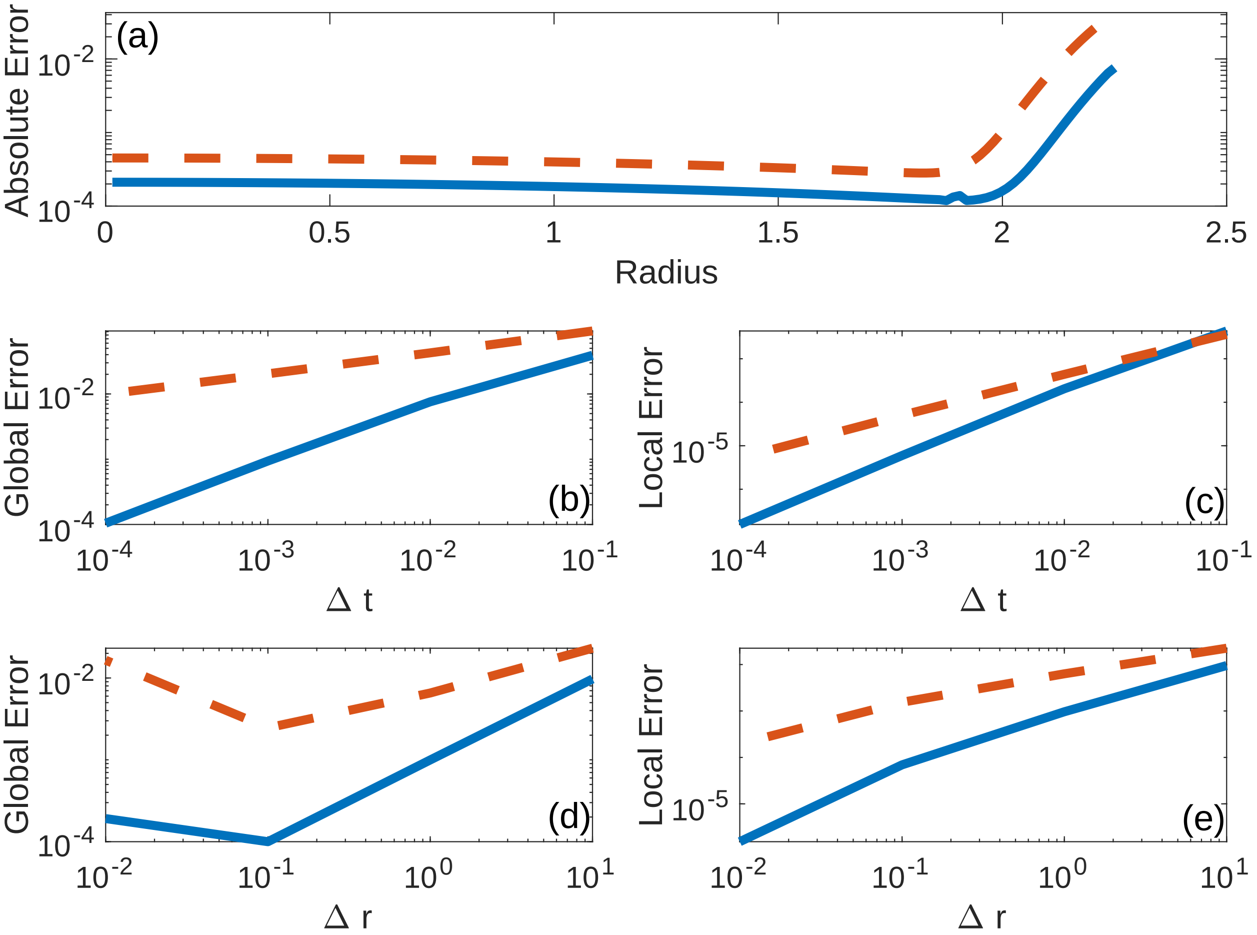

Figure F: *Convergence analysis via method of manufactured solutions.* Dashed (red) curve indicates $v(r,t)$ and solid (blue) curves indicate $u(r,t)$. **(a)** Absolute spatial error for $\Delta t = 0.01$ and $\Delta r = 0.015$ for $t \gg 0$. **(b)** Absolute global error for $\Delta t = 0.1, 0.01, 0.001, 0.0001$. **(c)** Absolute local error for $\Delta t = 0.1, 0.01, 0.001, 0.0001$ at $r = 0.5$. **(d)** Absolute global error for $\Delta r = 10, 1, 0.1, 0.01$. **(e)** Absolute local error for $\Delta r = 10, 1, 0.1, 0.01$ at $r = 0.5$.

S4 Supplementary Tables

symbol	description	unit	Type I	Type II	Type III	Type IV
a	tumor proliferation rate	day^{-1}	0.25 [4, 5]	0.125[4]	0.025[6]	0.25 [6]
D_{T^*}	tumor diffusion constant	cm^2/day	1e-5 [7]	2e-5 [7]	1e-4 [8]	1e-4 [8]
u^*	tumor diffusion threshold	cells/cm^3	1.2e8	2.39e7	2.39e6	2.39e6
$1/b$	tumor carrying capacity	cells/cm^3	2.39e8 [9]			
D_C	CAR T cell diffusion constant	cm^2/day	1.38e-2 [10]			
d	maximum lysis rate	day^{-1}	1.8 [1]			
s	E:T ratio for half-maximal lysis	unitless	0.55 [1]			
l	CAR T cell cooperativity	unitless	1.7 [1]			
j	maximum CAR T cell proliferation rate	day^{-1}	0.48 [1]			
k	lysis rate for half-maximal CAR T cell proliferation	$\text{cells}^2/\text{day}^2$	2.4e7 [1]			
q	CAR T cell exhaustion rate	$\text{cells}^{-1}\text{day}^{-1}$	1.84e-9 [1]			
m	CAR T cell death rate	day^{-1}	0.35 [1]			

Table A: Parameter values used in numerical simulations of the 4 tumor types described in section 2.2. The source used to determine a reasonable value for each parameter is indicated in brackets next to each value. A fraction of the tumor carrying capacity was chosen as the tumor diffusion threshold for each tumor type to obtain characteristic, qualitative behavior. For parameter values below the horizontal line (tumor carrying capacity and CAR T cell related parameters), the same values were used when simulating all tumor types.

symbol	description	unit	Zhao et al.	Skovgard et al.	range tested
a	tumor proliferation rate	day^{-1}	0.08	0.2	
D_{T^*}	tumor diffusion constant	cm^2/day	8e-5	1e-4	
u^*	tumor diffusion threshold	cells/cm^3	2.39e-7	2.39e-7	
$1/b$	tumor carrying capacity	cells/cm^3	2.39e8	2.39e8	
D_C	CAR T cell diffusion constant	cm^2/day	1.38e-2	1.38e-2	
d	maximum lysis rate	day^{-1}	12	19.25	0.1-25
s	E:T ratio for half-maximal lysis	unitless	0.25	0.2	0.1-10
l	CAR T cell cooperativity	unitless	1.36	1.24	0.1-4
j	maximum CAR T cell proliferation rate	day^{-1}	0.6	0.9	0.1-1
k	lysis rate for half-maximal CAR T cell proliferation	$\text{cells}^2/\text{day}^2$	2.05e7	2.05e7	
q	CAR T cell exhaustion rate	$\text{cells}^{-1}\text{day}^{-1}$	1.6e-9	1.6e-9	
m	CAR T cell death rate	day^{-1}	0.293	0.293	

Table B: Estimated model parameters to fit model output to murine study data. The range of values allowed for a given parameter in the initial run of the iterative search algorithm is included in the final column. For parameters that were fixed, rather than estimated, the final column is blank.

References

[1] Katherine Owens and Ivana Bozic. Modeling car t-cell therapy with patient preconditioning. *Bulletin of Mathematical Biology*, 83(5):1–36, 2021.

[2] J. Crank and P. Nicolson. A practical method for numerical evaluation of solutions of partial differential equations of the heat conduction type. *Proc. Camb. Phil. Soc.*, 43(1):50–67, 1947.

[3] Elizabeth A Eisenhauer, Patrick Therasse, Jan Bogaerts, Lawrence H Schwartz, Danielle Sargent, Robert Ford, Janet Dancey, S Arbuck, Steve Gwyther, Margaret Mooney, et al. New response evaluation criteria in solid tumours: revised recist guideline (version 1.1). *European journal of cancer*, 45(2):228–247, 2009.

[4] Eun Bi Ryu, Jung Min Chang, Mirinae Seo, Sun Ah Kim, Ji He Lim, and Woo Kyung Moon. Tumour volume doubling time of molecular breast cancer subtypes assessed by serial breast ultrasound. *European radiology*, 24(9):2227–2235, 2014.

[5] Piyush Nathani, Purva Gopal, Nicole Rich, Adam Yopp, Takeshi Yokoo, Binu John, Jorge Marrero, Neehar Parikh, and Amit G Singal. Hepatocellular carcinoma tumour volume doubling time: a systematic review and meta-analysis. *Gut*, 70(2):401–407, 2021.

[6] Toshinori Yamashita and Takeo Kuwabara. Estimation of rate of growth of malignant brain tumors by computed tomography scanning. *Surgical neurology*, 20(6):464–470, 1983.

[7] Jared A Weis, Michael I Miga, Lori R Arlinghaus, Xia Li, Vandana Abramson, A Bapsi Chakravarthy, Praveen Pendyala, and Thomas E Yankeelov. Predicting the response of breast cancer to neoadjuvant therapy using a mechanically coupled reaction–diffusion model. *Cancer research*, 75(22):4697–4707, 2015.

[8] Kristin R Swanson, Carly Bridge, JD Murray, and Ellsworth C Alvord Jr. Virtual and real brain tumors: using mathematical modeling to quantify glioma growth and invasion. *Journal of the neurological sciences*, 216(1):1–10, 2003.

[9] Ugo Del Monte. Does the cell number 109 still really fit one gram of tumor tissue? *Cell cycle*, 8(3):505–506, 2009.

[10] Matthias Mulazzani, Simon P Fräßle, Iven von Mücke-Heim, Sigrid Langer, Xiaolan Zhou, Hellen Ishikawa-Ankerhold, Justin Leube, Wenlong Zhang, Sarah Dötsch, Mortimer Svec, et al. Long-term in vivo microscopy of car t cell dynamics during eradication of cns lymphoma in mice. *Proceedings of the National Academy of Sciences*, 116(48):24275–24284, 2019.
