# Supplementary material for: Spatiotemporal dynamics of tumor–CAR T-cell interaction following local administration in solid cancers
Source: PLoS Comput Biol. 2025 Jun 3;21(6):e1013117. doi: 10.1371/journal.pcbi.1013117 (PMC12165384; doi:10.1371/journal.pcbi.1013117)
Supplement: Attachment — Submitted filename: Review of PCOMPBIOL-D-24-01849.docx [file pcbi.1013117.s003.docx]

Review of
”Spatiotemporal dynamics of tumor- CAR T-cell interaction following local administration in solid cancers”

In this paper, the authors introduce a straight-forward model of how a solid tumor reacts to treatment using immunotherapy. Their contribution lies in studying how mode of CAR-T cell injection affect the long-term outcome of a patient, contrasting two approaches. The first approach is when the CAR T-cells approach the tumor from the outside, working their way in to eradicate the tumor. The second approach is injecting the CAR-T population straight into the center of the tumor, working their way out. The authors are able to draw some compelling conclusions based on their simulation work, and conduct a limited parameter fitting with data based on mouse models.

Some quite drastic assumptions are made in order to make the model easier to study. First and foremost, radial symmetry is assumed, which turns out to be of chief importance in their computational approach. Secondly, there are no other interactions taking place between the cancer cells and the immune cells than immunological eradication of cancer cells. This means that the microenvironment and different taxis are not taken into consideration.

Reading this paper as an applied mathematician, I found it easy to follow and enjoyable, and I would recommend it for publication. The scope is clear, the limitations of the model are sufficiently addressed and the suggestion for further developments are sound. However, I have spotted some minor mistakes, and I have some suggestions regarding the structure of the paper that would make it even easier to follow. I will thus provide some comments on a chapter-by-chapter basis.

1. **Abstract**
   1. The sentence beginning with “Our results suggest that locally…” runs for too long and is a bit difficult to parse. Consider rephrasing.
2. **Introduction**
   1. On row 9-11, the terms “endurance”, “specificity” and “trafficking” are introduced, but with no further explanation given within the text. This might be fine if the article is written with an audience of mainly immunologists in mind, but I had to look up these terms.
   2. There is a broken reference on row 23.
   3. Later on (row 40-41), the basic structure of a reaction-diffusion model is explained in some sketchy detail, making me believe that the target audience are people unfamiliar with such concepts. I will have future comments on this, pertaining to the structure of the paper.
   4. Also on row 41-42, I would like to add that a reason that so much work on gliomas has been done is precisely because standard Fisher-esque reaction-diffusion models have proven insufficient in modelling these tumors complex morphology. Could be worth a mention.
   5. The sentences starting with “However, the post-injection…” on row 47 and ending on row 51 are concise and precisely written, giving the reader a clear view of the authors’ mission statement. Nice!
3. **Results**
   1. My first structural gripe with the paper is that results are presented *before* the mathematical model is discussed in greater detail. I found this very disorienting; even if the model itself is not the main focus of the paper, having it written down as a clear system of equations before we start discussing results from your simulations and inference would be of great aid. I would reshuffle the order of the chapters, putting “Materials and Methods” before result.
   2. On row 66, I would like the term “non-diffusively” to be clarified. Knowing the model, I think that the authors try to emulate the familiar “go-or-grow” dynamics observed in many tumor types, but since the model has not yet been properly introduced at this point, I was left wondering what they were talking about. Tumors can migrate by many different means; “non-diffusively” is too vague of a term.
   3. The formulation “We model tumor growth logistically…” on row 66-68 is needlessly wordy for explaining such a basic concept; I found it more confusing rather than erudite.
   4. The classification of tumors into four groups feel a bit arbitrary given the number of parameters considered. Some type of sketch or table could be of use, I provide an example below.

   5. Figure 1A does not aid me in understanding the model better – maybe if the equations were already introduced at this point it would have been better. 1B is very clear, however.
   6. The textbox belonging to Figure 2 is difficult to parse; I think it would be better to make a table out of the parameter values.
   7. In Figure 3, the description for panel A-B and C-D are flipped.
   8. For both Figure 3 and Figure 4, you do not mention type II tumors at all, making me wonder why this type was given any type of special consideration in the first place.
   9. Regarding Figure 6 and the accompanying text, I wish that the authors would have been clearer with how the classes “stable disease, partial response” etc are distinguished. The authors make a reference to the Choi criteria, but it would have been nice to have more on this in the actual text and not in the supplementary material.
   10. The same hold true for the “CAR T-cell treatment fails if CAR T cells move too slowly” section; it feels like many of the figures from the supplementary material could have been useful to display here.
4. **Discussion**
   1. I have very little to add here; almost every thought I had reading the manuscript is addressed and presented in a clear fashion. Two things that come to mind, that might be of interest:
      - Did you ever consider adapting an agent-based model? While the computational complexity might become infeasible for large populations, I think it would be interesting to contrast a 3D cellular automata with your PDE-based approach.
      - As it stands, your PDE formulation is still very “mean field”, which is of course a much better approximation of reality than well-mixed ODE models but it still leaves things such as mechanical cell-to-cell interactions out. For an introduction on how to formulate macroscopic models where cell adhesion/repulsions are taken into account, you can read “An interacting particle system modelling aggregation behavior: from individuals to populations” by Oelschläger et al. It might be an interesting read for future developments of this model.
   2. Overall, the discussion was enjoyable to read.
5. **Materials and methods**
   1. As stated earlier, I think this entire chapter should be moved to before the “Results” chapter. As for the model itself, it is fairly straightforward and based on sound principles. I only have a few reflections, that might warrant some future consideration.
   2. The choice to make the diffusion coefficient binary is probably based on the “go-or-grow” principle expressed by many cancer cell populations. However, having such a discontinuity is risky from a computational perspective. In the supplementary material, we can see that you take this into account by splitting the space into “low u” and “high u” segments, treating the joints as boundaries. This is however only possible due to the assumption of radial symmetry. Have you considered how well your model, and the numerical schemes, would adapt to other geometries?
   3. The assumption that CAR T-cells freely diffuse throughout the tumor is okay if the CAR T-cells are small relative to the tumor, but it might be problematic if this is not the case. See my remark above on the “mean fieldedness” of the model.
   4. You keep track of the population of exhausted CAR T-cell, but to my understanding they have no other bearing on the model other than contributing to the total CAR T-cell count. Is v(x,t) the density of the effector CAR T-cells? Do the exhausted cells have any effect on the system?
   5. For the parameter inference, how much stock are you putting into the values you’ve inferred for the four parameters you did not fix in your inference? How do you think your findings are biased by for example fixing the carrying capacity and the diffusion threshold a priori? Was the point of the parameter estimation to be more of a proof of concept that this model can be fit to data?

To close out, I found this paper to be an enjoyable read. Taking these remarks into account, I find the paper suitable for publication.
