# Supplementary material for: Spatiotemporal dynamics of tumor–CAR T-cell interaction following local administration in solid cancers
Source: PLoS Comput Biol. 2025 Jun 3;21(6):e1013117. doi: 10.1371/journal.pcbi.1013117 (PMC12165384; doi:10.1371/journal.pcbi.1013117)
Supplement: Attachment — Submitted filename: ResponseToReviewers.docx [file pcbi.1013117.s004.docx]

Thank you for your thoughtful handling of our manuscript, “Spatiotemporal dynamics of tumor - CAR T cell interaction following local administration in solid cancers.” We appreciate the time, attention to detail and expertise of the reviewers as they provided insightful feedback. We truly believe these edits have improved the work. Please see our point-by-point responses to reviewer comments below.

Reviewer #1:

In this article by Owens, Rahman, and Bozic, the authors present a mathematical model to study two different routes of administration of CAR T cells in solid tumors. They use previously published preclinical data and prior literature to ground their model in data and to study different possible therapeutic outcomes. The manuscript is well written, well motivated, and is likely to be of interest to a wide audience in the fields of mathematical biology, cancer research, and CAR T cells.

The authors principal conclusion is that “We demonstrate that the model can recapitulate tumor and CAR T-cell data from small imaging studies of local administration of CAR T cells in mouse models.”, and that “locally administered CAR T cells will be most successful against slowly proliferating, highly diffusive tumors.” I have concerns about both of these conclusions, namely 1) their model is fit to imaging data, and therefore does not recapitulate the dynamics per say as much as reproduce them, and 2) the efficacy against highly diffusive tumors may be an artifact of the diffusion thresholding in their model. However, there are plenty of insights from their model that can be well justified and supported as conclusions.

Thank you for the kind evaluation of the writing and significance of the paper, and for providing thoughtful feedback to improve the manuscript.

We completely agree with the reviewer’s suggestion and have replaced “recapitulate” with “reproduce” throughout the text. We also updated the first sentence in the relevant subsection of the results to say, “*After establishing the general behavior of our model, we verified that the model could be fit to data from early imaging studies in mice*”

We had not considered that the efficacy against highly diffusive tumors may be an artifact of the diffusion thresholding. In response to this concern, we investigated the effect of removing the diffusion threshold from the model. First, we re-ran the code generating initial tumor conditions and tumor growth in the absence of treatment using the same set of parameter values, $a$, $b$, and $D_{T}$, for tumor Types 1-4 but setting the diffusion threshold $u* = 0$. We used the resulting simulations to generate an analogous Figure to Figure 2 from the manuscript, included below.

Next, we applied intratumoral treatment to these four tumors. We observed the same four general treatment outcomes from intratumoral injection of CAR T cells as models with u*>0, though the exact ranges of CAR T-cell doses for which specific outcomes occur shifts.

We identified the minimal effective dose of intratumoral CAR T-cell therapy against these tumor types for a tumor of detectable diameter 2 cm and found that CAR T-cells are still most effective against tumors with low proliferation and high diffusivity (type 3). We find a relationship between average tumor density and the efficacy of CAR T-cell therapy both with u*=0 and u*>0. See the plot below showing the minimum CAR T-cell dose for eradication with intratumoral injection vs. density of the detectable tumor at the time of treatment. We have also plotted the minimum CAR T-cell dose for eradication against the ratio of proliferation rate, a, to the diffusion constant, $D_{T}$. In the case that u*=0 and for a fixed value of b, this ratio dictates the tumor density. We found that both initial density and ${a/D}_{T}$ can explain over 80% of the variance in minimum dose for complete response on a log-log scale (R^2 = 0.84 and R^2 = 0.83 respectively). In contrast, assuming the same detectable tumor diameter, total tumor burden and volume doubling time explains less than 40% of the variance in minimum dose (R^2 = 0.38 and R^2 = 0.064 respectively).

I have a few significant concerns regarding the modeling:

Why do the authors threshold diffusion? This is a major assumption in the model that is not consistent with previous PDE modeling in this area, and is explained or justified at all.

Thank you for drawing our attention to the need to better justify this choice in the manuscript. Our main motivation for using the thresholded diffusion term in our model was to incorporate two phases of tumor growth, an initial stationary growth phase and an invasive phase, into a unified model. We have now included the following sentences at the beginning of the “Model Equations” section to better explain and justify this choice:

*“We use a density-dependent threshold for diffusion to reflect two modes of tumor growth in a unified model: a stationary phase, in which the tumor grows more dense but does not expand, and a diffusive phase, in which tumor cells both proliferate and spread. These two phases are rooted in observed phenotypic differences in the motility and proliferative capacity of cancer cells [73]. The ``go-or-grow" hypothesis postulates that external factors such as low oxygen levels, chemoattractants and the rigidity of the extracellular matrix may cause cancer cells to switch between a less motile, highly proliferative state and a more invasive state [74]. In this simple initial model, we do not explicitly model these external factors, but assume that the transition to a more invasive state occurs when the tumor density at each spatial location surpasses a critical value.”*

Beyond the biological motivation, with thresholded diffusion, we have the added benefit that using a threshold u* > 0 keeps the tumor bounded away from the edge of our computational domain. This avoids having to deal with leakage or impose a Neuman boundary condition on tumor density. Having a clearly defined tumor surface well-within the computational domain is also helpful for determining where to initiate the CAR T cells for intracavitary administration. We have added the following to the Numerical Simulations subsection of the Materials and Methods, so it now reads:

“*The extent of the spatial domain was selected based on the initial tumor size and anticipated tumor growth such that the tumor would not reach within 1 cm of the edge of the domain. This is only possible when using a threshold u* > 0 for diffusion. The diffusion threshold keeps the tumor bounded away from the edge of a sufficiently large computational domain, avoiding leakage or having to impose a Neuman boundary condition.* ”

With the diffusion threshold, we also have another parameter that gives us added flexibility for easily generating a wide variety of tumor profiles. When we use u*=0 for the same values of $a$ and $D_{T}$above, we see a less varied range of initial tumor profiles. The densest tumor type is slightly less dense and the detectable part of the least dense tumor type becomes significantly more dense. We could achieve a slightly wider range of tumor profiles with u*= 0 by exploring more varied ratios of ${a/D}_{T}$, but using a threshold for diffusion was a convenient alternative.

The authors glaze over the exhausted cell compartment, and provide no rationale for its inclusion or the key parameter (q) in determining the fraction. Also, exhausted cells are not graphed or discussed in detail. So why include them?

We thank the reviewer for pointing out this gap in our write-up! The reviewer is correct that in our current model the exhausted CAR T cells do not interact with tumor cells, so they are not necessary to track for predicting tumor outcomes. However, we found that including the exhausted CAR T-cell compartment produces CAR T-cell kinetics with the expected pattern of expansion and contraction and improved the fit to the CAR T-cell data from imaging studies. Considering exhausted cells in the infusion product also brought our results more in line with the reality that current CAR T-cell treatment of solid tumors have been largely unsuccessful. We have added the following figure to the supplement, to illustrate how the exhausted cells contribute to the total CAR T-cell count over time for the examples from Figure 5. We also added the following sentences to the discussion:

*“We found that accounting for exhausted CAR T-cells in model simulations improved the fit to CAR T-cell data from a study that tracked CAR T-cell kinetics. The possibility of exhausted cells in the CAR infusion product also affects the minimum CAR T dose necessary to achieve a given outcome, bringing our results more in line with the reality that current CAR T-cell treatment of solid tumors have been largely unsuccessful [3].”*

We appreciate that the reviewer’s comment prompted us to look into the exhausted compartment more deeply, because we caught an error in the code for re-dimensionalizing the exhausted cells when generating Figure 5 in our original submission. With the correct dimensionalization, both examples had a higher peak number of total CAR T-cells. For the Skovgard et al. example, this meant the model simulations no longer agreed well with the CAR T-cell data. We have refitted the CAR T-cell parameters matching the total cell count when using the correct count for exhausted cells, and have replaced panels E and F in Figure 5.

Similarly, the D function (after equation 3b) is complex and not motivated at all. F2 appears to be a very interesting and complex term. Please explain to the reader.

Thank you for the suggestion to explain the details of the forcing functions in more detail. We have restructured the paper so that the model equations are explained prior to presenting the results of numerical simulations. In the “Model Equations” section, we now explain the tumor cell lysis term D as follows:

*“Tumor cells are killed by CAR T cells at a rate determined by the ratio of CAR T-cell to tumor density in a given location. Tumor cells can be killed at a maximum rate of $d$, with the half-maximal killing rate attained at the CAR T-cell to tumor ratio, $s$. The exponent, $l$, dictates the slope of the saturation curve, with larger values leading to increasingly ``switch-like" behavior. This phenomenological functional form was proposed by de Pillis et al. in a model of adoptive cellular therapy and found to best fit cytotoxicity assay data compared with mass action or traditional Michaelis-Menten kinetics [76].”*

In the same section, we also explain the CAR T cell forcing function F2 in more detail:

“*The CAR T-cell proliferation term was also inspired by the model of tumor-T-cell interactions studied by de Pillis et al. [76]. With this functional form, CAR T-cell proliferation depends on cell lysis,*$D(x,t)$*. The maximum rate of cell proliferation is* $j$*, with half-maximal proliferation occurring when* $D^{2}=k$*. Using a saturating term for the CAR T-cell proliferation rate imposes an upper limit, which is reasonable due to the biological constraints on cell division. This term differs from the recruitment term used by Owens and Bozic [45] because we do not include endogenous effector cells in this model. CAR T-cell density also decreases at rate* $m$ *due to cell death and at rate* $q$ *due to exhaustion upon repeated interaction with tumor cells.”*

I think the assumption of uniform CAR T cell density on the tumor surface (even in spherical symmetry) is far too simplified, but I do not insist it be changed. Consider providing much more justification for this.

We fully agree with the reviewer that this is a highly simplified choice for the geometry of CAR T cells in the case of intracavitary administration. We felt justified using a symmetrical initial density of CAR T cells on the tumor surface because imaging data from Mullazzani et al. [75] indicates that CAR T cells rapidly disperse across a tumor. Out of many possibilities, we chose to use a uniform density to avoid introducing additional parameters. We have now added the following sentence to the section “Intracavitary delivery can delay tumor progression” to justify this choice:

*“To simulate intracavitary delivery of CAR T cells, we initiate the system with one of the four final tumor profiles in Fig. 2A, and a thin layer of CAR T cells uniformly concentrated on the surface of the tumor. Using a uniform, symmetrical initial distribution of CAR T cells is a heavily simplified, numerically expedient way to reflect the evidence from imaging studies showing that CAR T cells rapidly disperse across solid tumors [75].”*

Why do the authors present 4 tumor growth archetypes but present only 3 in figures 3,4? Confusing.

Thank you for pointing out this confusion. We have updated Figures 3 and 4. Now Figure 3 contains 4 examples illustrating complete response, partial response, stable disease and progressive disease outcomes and each tumor archetype is used for one of the examples. In Figure 4 we still include only 3 examples because a complete response was not achieved for tumors of diameter 2 cm treated with intracavitary delivery of CAR T cells. We also updated the inset panels showing tumor diameter over time for each example to note the relevant outcome.

I think the manuscript would benefit greatly from a discussion about the parameters and their meanings, particularly the CAR parameters. Can the tumor growth/diffusion and CAR dynamics reveal more specific/quantitative relationships between the parameters? Also, the 20% reduction in parameters to model ‘reduced efficacy in solid tumors’ is important and not studied or motivated.

We appreciate this suggestion from the reviewer. In the revised structure of the paper, the “Parameter Estimation” section now comes earlier. We expanded the sections explaining the forcing functions F1 and F2 in more detail as mentioned above. To highlight the relationship between CAR dynamics and parameter values, we now include the sensitivity analysis in the main text as Figure 8. We have also included a panel in the new supplementary Figure S3 illustrating the relationship between the minimum intratumoral CAR T-cell dose necessary for tumor eradication based on the ratio of tumor growth rate to the diffusion constant. We have also added the following sentence explaining our reasoning for the 20% reduction in CAR T cell parameter values:

*“The same approach was taken by Kara et al. [39] when adapting CAR T-cell models developed for blood cancers to solid tumors. We performed a local sensitivity analysis to study the effect of varying the CAR T-cell parameter values, which is discussed in the Results section.*”

Minor comments:
Check carefully for typos and latex issues. There are more than a few. (ex. T-cells vs. T cells).

We apologize for the plethora of typos in the original submitted version and have reviewed the final version for latex issues, particularly focusing on using T cell vs. T-cell throughout according to whether it is a noun or an adjective in the sentence as suggested in the NIH style guidelines.

Numerical values in caption of Figure 2 are cumbersome to read. They would be better as a table.

Thanks for pointing this out. We have removed these values from the caption and added Table 1.

I find figure 1A to be more confusing than helpful. Difficult to interpret the 4 arrows and dots, and the complex term at the top (jD(u,v)^2v/, …) is not explained in the caption or main text in sufficient detail to understand.

Based on both reviewers’ feedback, that Fig. 1A was not illuminating, we have removed the schematic. Instead the model equations are now explained in detail in the main text.

Can the variance across mice be shown in Figure 5? Authors state the model was fit to the mean value.

Thank you for the suggestion! We have added the error bars from Skovgard et al. to Fig. 5.

In vitro and ex vivo used to mean the same thing. Consider using a single term.

Thank you for catching our error. Throughout we now refer to ex vivo cells because we are consistently referring to cells that have been derived from patient samples.

The \cdot notation used in place of \nabla is unconventional and also not introduced or explained (equations 1,2).

We apologize for the latex error that arose when switching templates and appreciate that the reviewer caught this. We now include nablas in the equations, per standard notation.

Reviewer #2:

In this paper, the authors introduce a straight-forward model of how a solid tumor reacts to treatment using immunotherapy. Their contribution lies in studying how mode of CAR-T cell injection affect the long-term outcome of a patient, contrasting two approaches. The first approach is when the CAR T-cells approach the tumor from the outside, working their way in to eradicate the tumor. The second approach is injecting the CAR-T population straight into the center of the tumor, working their way out. The authors are able to draw some compelling conclusions based on their simulation work, and conduct a limited parameter fitting with data based on mouse models.

Some quite drastic assumptions are made in order to make the model easier to study. First and foremost, radial symmetry is assumed, which turns out to be of chief importance in their computational approach. Secondly, there are no other interactions taking place between the cancer cells and the immune cells than immunological eradication of cancer cells. This means that the microenvironment and different taxis are not taken into consideration.

Reading this paper as an applied mathematician, I found it easy to follow and enjoyable, and I would recommend it for publication. The scope is clear, the limitations of the model are sufficiently addressed and the suggestion for further developments are sound. However, I have spotted some minor mistakes, and I have some suggestions regarding the structure of the paper that would make it even easier to follow. I will thus provide some comments on a chapter-by-chapter basis.

We thank the reviewer for their careful reading of the manuscript, generous evaluation, and helpful suggestions. We are pleased with how these edits improved the paper.

**1.**	**Abstract**

1.	The sentence beginning with “Our results suggest that locally…” runs for too long and is a bit difficult to parse. Consider rephrasing.

Thank you for flagging this. We have now split this into two sentences:

*“Our results suggest that locally administered CAR T cells will be most successful against slowly proliferating, highly diffusive tumors. In our simulations, assuming equal detectable tumor diameters at the time of treatment, low average tumor cell density is a better predictor of treatment success than total tumor burden or volume doubling time.”*

**2.**	**Introduction**

**1.**	On row 9-11, the terms “endurance”, “specificity” and “trafficking” are introduced, but with no further explanation given within the text. This might be fine if the article is written with an audience of mainly immunologists in mind, but I had to look up these terms.

We appreciate this note on including the correct level of background for the PLOS Computational Biology audience and have now updated this sentence to remove jargon as follows.

*“In order to overcome the challenges presented by the complex biology of solid tumors, advances on three key fronts are being investigated: the specificity of CAR T cells [4] , meaning their ability to recognize all tumor cells without targeting healthy cells, the long-term persistence of CAR T cells in the body [5,6], and the ability of CAR T cells to reach the tumor site and infiltrate the tumor [7,8].”*

**2.**	There is a broken reference on row 23.

Thank you for catching this. We removed the hanging comma that was causing this error.

**3.**	Later on (row 40-41), the basic structure of a reaction-diffusion model is explained in some sketchy detail, making me believe that the target audience are people unfamiliar with such concepts. I will have future comments on this, pertaining to the structure of the paper.

Thank you for your perspective--we appreciate all the suggestions to better tailor the paper to the PLOS Comp Bio readership.

**4.**	Also on row 41-42, I would like to add that a reason that so much work on gliomas has been done is precisely because standard Fisher-esque reaction-diffusion models have proven insufficient in modelling these tumors complex morphology. Could be worth a mention.

Great point! We have added the following reference to this in the introduction:

*“Later models incorporating additional biological details like nutrient limitation [57-60] and non-uniform growth and migration among cells due to intrinsic and extrinsic factors [61-65], have built on this foundation to generate more realistic tumor growth patterns. ”*

And we added the following to the discussion:

“*The early, Fisher-esque reaction-diffusion models were unable to capture the complex morphology of gliomas observed in experimental and clinical data, motivating incorporation of further biological details. In future work, CAR T cells may be integrated into the numerous more advanced models that exist for gliomas.”*

**5.**	The sentences starting with “However, the post-injection…” on row 47 and ending on row 51 are concise and precisely written, giving the reader a clear view of the authors’ mission statement. Nice!

Thank you :)

**3.**	**Results**

**1.**	My first structural gripe with the paper is that results are presented *before* the mathematical model is discussed in greater detail. I found this very disorienting; even if the model itself is not the main focus of the paper, having it written down as a clear system of equations before we start discussing results from your simulations and inference would be of great aid. I would reshuffle the order of the chapters, putting “Materials and Methods” before result.

Thanks for this suggestion. We have reordered the sections accordingly and expanded the explanation of the model, and we think this improves the readability significantly.

**2.**	On row 66, I would like the term “non-diffusively” to be clarified. Knowing the model, I think that the authors try to emulate the familiar “go-or-grow” dynamics observed in many tumor types, but since the model has not yet been properly introduced at this point, I was left wondering what they were talking about. Tumors can migrate by many different means; “non-diffusively” is too vague of a term.

Thank you for pointing out this ambiguity. We have edited this sentence to remove the term “non-diffusively,” so it now reads, *“We use a density-dependent threshold for diffusion to reflect two modes of tumor growth in a unified model: a stationary phase, in which the tumor grows more dense but does not expand, and a diffusive phase, in which tumor cells*

*both proliferate and spread.”*

**3.**	The formulation “We model tumor growth logistically…” on row 66-68 is needlessly wordy for explaining such a basic concept; I found it more confusing rather than erudite.

We appreciate this feedback. In the updated Mathematical Model section we have simplified this sentence to, *“Thus, in our model tumor growth is logistic, with proliferation rate a and a local carrying capacity of 1/b for the density at each spatial point in the domain.”*

**4.**	The classification of tumors into four groups feel a bit arbitrary given the number of parameters considered. Some type of sketch or table could be of use, I provide an example below.

 We agree that four groups was quite an arbitrary choice, and the diagram provided by the reviewer is exactly how we arrived at this number! We initially chose four tumor types with two values of $a$ and two values of ($D_{T*}, u*$). However, the low $a$ , low ($D_{T*}, u*$) tumor was so slow growing that it would not be a reasonable candidate for CAR T cell therapy. Consequently we bumped both $a$ and ($D_{T*}, u*$) up to arrive at tumor type II. We have added the schematic suggested by the reviewer to Figure 2 as an inset to help orient the reader to how the tumor types are related.

**5.**	Figure 1A does not aid me in understanding the model better – maybe if the equations were already introduced at this point it would have been better. 1B is very clear, however.

Based on the unanimous reviewer feedback that Fig. 1A was not illuminating, we have removed the schematic. Instead, the model equations are now explained in detail in the main text.

**6.**	The textbox belonging to Figure 2 is difficult to parse; I think it would be better to make a table out of the parameter values.

Good point! We have removed these values from the caption and added Table 1.

**7.**	In Figure 3, the description for panel A-B and C-D are flipped.

Thank you for catching this typo. We have corrected this for the new Figure 3.

**8.**	For both Figure 3 and Figure 4, you do not mention type II tumors at all, making me wonder why this type was given any type of special consideration in the first place.

We appreciate how introducing the 4 tumor types and then including 3 examples in Figure 3 and 4 is confusing. In the revised manuscript we have updated Figures 3 and 4. Now Figure 3 contains 4 examples illustrating complete response, partial response, stable disease and progressive disease outcomes and each tumor archetype is used for one of the examples. In Figure 4 we still include only 3 examples because a complete response was not achieved for tumors with diameter 2 cm. Type II tumors are included both for completeness in covering the schematic suggested above, and we found it satisfying that this tumor archetype displays all four possible RECIST outcomes in response to the range of CAR T-cell doses tested in Figure 6.

**9.**	Regarding Figure 6 and the accompanying text, I wish that the authors would have been clearer with how the classes “stable disease, partial response” etc. are distinguished. The authors make a reference to the Choi criteria, but it would have been nice to have more on this in the actual text and not in the supplementary material.

Thank you for this suggestion. We have added the following sentences explaining the RECIST criteria so that the manuscript is more self-contained for the reader.

*“The RECIST criteria is a standardized set of guidelines widely used to assess the efficacy of cancer treatments against solid tumors. Under these guidelines tumor response is classified as complete response if all measurable lesions disappear, a partial response if measurable lesions decrease in diameter by at least 30%, or progressive disease if measurable lesions increase in diameter by 20% or new lesions form. Otherwise the response is deemed stable disease. ”*

To further clarify, we illustrated the classifications graphically above the outcome maps in the new Figure 6. We also moved Figure S4 to the main text as the new Figure 7 to reduce the references to the supplement in the results section.

**10.** The same hold true for the “CAR T-cell treatment fails if CAR T cells move too slowly” section; it feels like many of the figures from the supplementary material could have been useful to display here.

We appreciate the reviewer’s point about referring the reader to the supplementary figures so often. We have moved both Figure S4 illustrating different classifications based on the Choi criteria vs. the RECIST criteria for evaluating patient outcomes and Figure S5 illustrating the results of sensitivity analysis on the CAR parameters into the main text.

**4.**	**Discussion**

**1.**	I have very little to add here; almost every thought I had reading the manuscript is addressed and presented in a clear fashion. Two things that come to mind, that might be of interest:

§ Did you ever consider adapting an agent-based model? While the computational complexity might become infeasible for large populations, I think it would be interesting to contrast a 3D cellular automata with your PDE-based approach.

We agree that this would be interesting and that the problem would be well-suited to agent-based modeling! We could consider a 2D domain calibrated to single-cell imaging modalities such as imaging mass cytometry, like [Mongeon et al.](https://www.nature.com/articles/s41540-024-00419-4) have done for glioblastoma.

§ As it stands, your PDE formulation is still very “mean field”, which is of course a much better approximation of reality than well-mixed ODE models but it still leaves things such as mechanical cell-to-cell interactions out. For an introduction on how to formulate macroscopic models where cell adhesion/repulsions are taken into account, you can read “An interacting particle system modelling aggregation behavior: from individuals to populations” by Oelschläger et al. It might be an interesting read for future developments of this model.

Thank you very much for this suggestion!

**2.**	Overall, the discussion was enjoyable to read.

Thank you for the kind feedback.

**5.**	**Materials and methods**

**1.**	As stated earlier, I think this entire chapter should be moved to before the “Results” chapter. As for the model itself, it is fairly straightforward and based on sound principles. I only have a few reflections, that might warrant some future consideration.

We have made this change in structure and appreciate how it strengthens the paper.

**2.**	The choice to make the diffusion coefficient binary is probably based on the “go-or-grow” principle expressed by many cancer cell populations. However, having such a discontinuity is risky from a computational perspective. In the supplementary material, we can see that you take this into account by splitting the space into “low u” and “high u” segments, treating the joints as boundaries. This is however only possible due to the assumption of radial symmetry. Have you considered how well your model, and the numerical schemes, would adapt to other geometries?

We completely agree with the reviewer that the numerical methodology, specifically the use of a Crank-Nicolson finite difference scheme for this geometry only works due to the radial symmetry. The scheme will not scale up to generic geometries, and therefore we must employ finite element methods such as in Kara *et al.* [63] for more complex geometries, which we leave for a future study. In fact, we have begun preliminary development on an FEM scheme [Crupi, *UW MS Thesis*], however this work is still in the very early stages. To let the reader be aware of this particular shortcoming of the numerical scheme we have added the following line to the numerical analysis section: *``The use of Crank-Nicolson finite difference methods on an equation with a step-function diffusivity is made possible due to the radial symmetry of the domain. For more complex geometries it will be necessary to employ finite element methods, which we leave for a future study."*

**3.**	The assumption that CAR T-cells freely diffuse throughout the tumor is okay if the CAR T-cells are small relative to the tumor, but it might be problematic if this is not the case. See my remark above on the “mean fieldedness” of the model.

This is such an important consideration and certainly points towards developing a less “mean field” approach, such as an agent based model. That would be a great setting to incorporate tumor phenotypes like varied degrees of cell clumping, which can hinder T cell penetration into gliomas. Thank you for the suggestion and food for thought!

**4.**	You keep track of the population of exhausted CAR T-cell, but to my understanding they have no other bearing on the model other than contributing to the total CAR T-cell count. Is v(x,t) the density of the effector CAR T-cells? Do the exhausted cells have any effect on the system?

We thank the reviewer for pointing out this gap in our write-up too. The same response as to reviewer 1 applies here:

The reviewer is correct that in our current model the exhausted CAR T cells do not interact with tumor cells, so they are not necessary to track for predicting tumor outcomes. However, we found that including the exhausted CAR T-cell compartment produces CAR T-cell kinetics with the expected pattern of expansion and contraction and improved the fit to the CAR T-cell data from imaging studies. Considering exhausted cells in the infusion product also brought our results more in line with the reality that current CAR T-cell treatment of solid tumors have been largely unsuccessful. We have added the following figure to the supplement, to illustrate how the exhausted cells contribute to the total CAR T-cell count over time for the examples from Figure 5. We also added the following sentences to the discussion:

*“We found that accounting for exhausted CAR T-cells in model simulations improved the fit to CAR T-cell data from a study that tracked CAR T-cell kinetics. The possibility of exhausted cells in the CAR infusion product also affects the minimum CAR T dose necessary to achieve a given outcome, bringing our results more in line with the reality that current CAR T-cell treatment of solid tumors have been largely unsuccessful.”*

We appreciate that the reviewer’s comment prompted us to look into the exhausted compartment more deeply, because we caught an error in the code for re-dimensionalizing the exhausted cells when generating these plots in our original submission. With the correct dimensionalization, both examples had a higher peak number of total CAR T-cells. For the Skovgard et al. example, this meant the model simulations no longer agreed well with the CAR T-cell data. We have refitted the CAR T-cell parameters matching the total cell count when using the correct units for exhausted cells, and have replaced panels E and F in Figure 5.

**5.**	For the parameter inference, how much stock are you putting into the values you’ve inferred for the four parameters you did not fix in your inference? How do you think your findings are biased by for example fixing the carrying capacity and the diffusion threshold a priori? Was the point of the parameter estimation to be more of a proof of concept that this model can be fit to data?

Thank you for these clarifying questions. Our goal in tuning the model to reproduce data from mouse studies was to check that it can produce reasonable results and as you suggest, serve as a proof of concept that, given more data, we could perform a more comprehensive parameter estimation. We have added the following sentences clarifying this in the results section:

*“These two examples provide proof-of-concept that the model can be fit to data from imaging studies. As additional data becomes available, a more robust parameter estimation procedure could be developed to better quantify CAR T cell-tumor dynamics.”*

To close out, I found this paper to be an enjoyable read. Taking these remarks into account, I find the paper suitable for publication.
